# Osteomyelitis of Scapula with Secondary Septic Arthritis of Shoulder Joint in a Six Month Old Child

**DOI:** 10.5704/MOJ.1303.001

**Published:** 2013-03

**Authors:** Sanjay Meena, Mohammed Tahir Ansari

**Affiliations:** Department of Orthopaedics, All India Institute of Medical Sciences, New Delhi, India; Department of Emergency Medicine, All India Institute of Medical Sciences, New Delhi, India

## Abstract

We report a case of delayed diagnosis of osteomyelitis of the scapula extending to glenoid, with secondary septic arthritis of glenohumeral joint in a 6-month-old female infant. We performed urgent arthrotomy of the shoulder joint through an anterior approach with drainage of pus from the scapula through a posterior approach and prescribed intravenous antibiotics. Diagnosis was delayed as the patient initially presented with pneumonia, for which she was treated in the neonatal intensive care unit; at that time, all medical efforts were directed toward saving her life. Had the diagnosis been made earlier, septic arthritis of the shoulder might have been prevented. Diagnosis of septic shoulder arthritis is rare and difficult, requiring a high index of suspicion.

**Key Words:**

septic arthritis, infection, scapular osteomyelitis

## Introduction

Osteomyelitis of the scapula is a rare clinical entity^1^. Acute septic arthritis of the shoulder in children typically occurs as a complication of osteomyelitis of the proximal humeral metaphysis^2^. We report a case of delayed diagnosis of scapular osteomyelitis in a 7-month-old female infant, which initially involved the inferior angle of the scapula, extending up to the glenoid. Subsequently osteomyelitis extended to the entire scapula and extended to glenohumeral joint causing secondary septic arthritis of the joint. The diagnosis of septic shoulder is uncommon and difficult, and requires a high index of suspicion 3.

## Case Report

A 6-month-old female infant was admitted to the paediatrics ward with fever and difficulty in breathing. Lungs were clear to auscultation, but based on clinical suspicion, she was diagnosed with pneumonia and we began to administer a course of intravenous ampicillin. On day two following admission, the patient’s condition deteriorated and she was transferred to the neonatal intensive care unit (NICU).

**Fig. 1:**
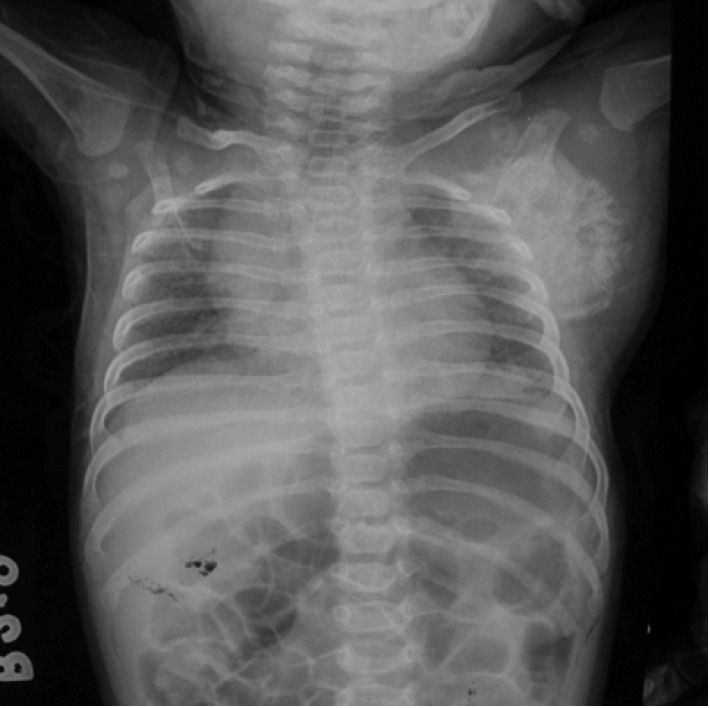
: Anteroposterior radiograph of the right shoulder showing obvious destruction of the glenoid and the lateral border of the scapula with normal humeral head.

**Fig. 2:**
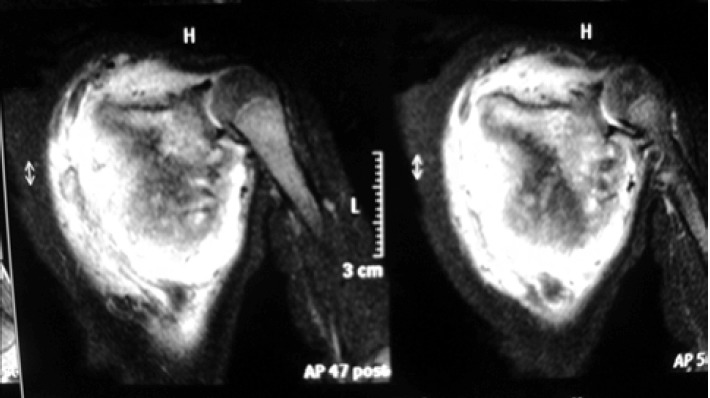
: Magnetic resonance image showing involvement of entire scapula and glenohumeral joint.

**Fig. 3:**
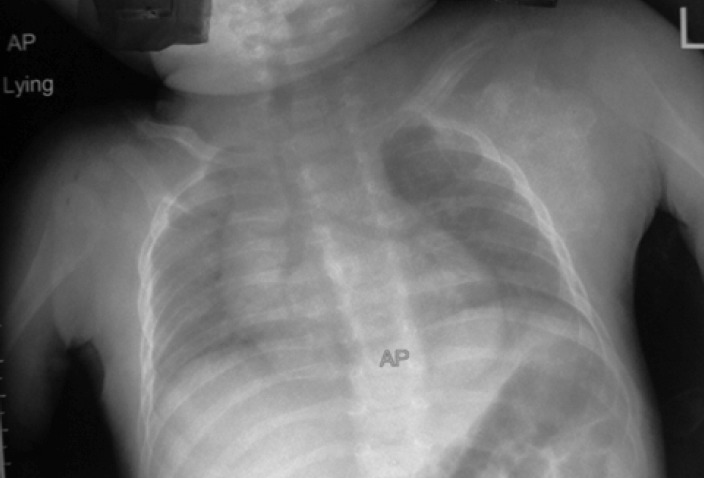
: Initial anteroposterior radiograph of the chest and both shoulders shows destruction of the glenoid and the lateral border of the scapula.

Subsequently, pseudo paralysis of the right upper limb was noted, accompanied by swelling of the right scapula and right shoulder associated with pain and aggravated by shoulder movement. An anteroposterior radiograph of the right shoulder showed osteomyelitis involving entire right scapula (Fig 1). Magnetic resonance imaging (MRI) confirmed the diagnosis (Fig 2). The patient’s leucocyte count, erythrocyte sedimentation rate (ESR) and C-reactive protein (CRP) were high (leucocyte count, 14,260 cells/mcL; neutrophils, 68.9%; lymphocytes, 19.4%; ESR, 35 mm/hr; and CRP 45 mg/L). Blood cultures were obtained, but no infectious organisms were isolated. Cultures from the lung infection grew Staphylococcus epidermidis. Retrospectively examination of previous chest radiographs revealed evidence of osteomyelitis over the inferior angle of the right scapula, extending to the glenoid (Fig 3). With the patient under general anaesthesia, we performed a right shoulder arthrotomy including joint incision and drainage of an abscess on the posterior aspect of the shoulder (drained posteriorly) in addition to drainage and irrigation of the joint through an anterior approach. Although the humeral head appeared to be healthy, other operative findings included scapular and glenoid erosions and destruction of the articular glenoid cartilage. The wound was closed over a drain, which was removed after 48 hours. Drainage was sent for culture and sensitivity tests, but no organism was detected.

On the second postoperative day, the patient’s fever was down and shoulder joint motion improved significantly in the second postoperative week. Intravenous Augmentin (amoxicillin and clavulanate) was prescribed for 2 weeks and then changed to Augmentin p.o. for 4 weeks. Postoperatively, laboratory values were as follows: CRP, 5.5 mg/L; leucocyte count, 8,300 cells/mcL; neutrophils, 47%; and lymphocytes, 54%. The patient was subsequently discharged and followed up on an outpatient basis. At the last follow-up visit, five months after discharge, the patient had regained excellent range of motion in the right shoulder except for some limitations in above-head abduction (> 90 degrees but unable to completely abduct).

## Discussion

Acute septic arthritis results from bacterial invasion of a joint space, which can occur through haematogenous spread, direct inoculation from trauma or surgery, or contiguous spread from an adjacent site of osteomyelitis or cellulitis. The diagnosis of septic arthritis can be difficult, especially in neonates and infants, as they present with minimal clinical findings such as fever, swelling or pain. In addition, multifocal involvement, which is not apparent at presentation, is common in this age group.

Frequently infection spread via haematogenous route from a distant primary site of entry (e.g., respiratory, ear, nose, throat or skin). In the present case, the patient first presented with pneumonia and infection spread to the bone and joint from the respiratory system. The patient had mild pneumonia, as the lung fields were clear; instead, the high grade fever and worsening patient condition stemmed more from osteomyelitis/septic arthritis. Septic arthritis or osteomyelitis should be suspected in infants who present with fever, unexplained limp, reluctance to use the limb, musculoskeletal pain with or without local bone or joint tenderness, and bone or joint swelling.

Most patients with septic arthritis and osteomyelitis of the lower extremities present with a limp or an inability to bear weight. In the upper extremities, decreased use of the shoulder associated with discomfort, pain or pseudoparalysis is the most common presenting complaint for septic arthritis or osteomyelitis in the shoulder region. Younger children often do not present with these classic symptoms. Septic arthritis of the shoulder joint secondary to osteomyelitis of the scapula is uncommon disease but requires a high index of suspicion.

In the present case, retrospective examination of chest x rays revealed bone destruction over the inferior angle of the right scapula extending to the glenoid along the lateral scapular border. Had it been detected at the time of the initial chest radiograph, appropriate treatment could have been started earlier with better outcome. In the infant, transphyseal vessels are patent and infection may spread to the adjacent joint causing septic arthritis. Degree of delay prior to treatment initiation is the most important prognostic factor. Late diagnosis may lead to damage in the articular surfaces. Involvement of the proximal humerus in infants and young patients may lead to the deformation of the humeral head, with shortening of the humerus. Associated infectious focus, more prevalent in the paediatric population as in the present case, adds to the difficulty in diagnosis. Early diagnosis and treatment can prevent articular damage and preserve the range of joint motion. Treatment should include intravenous antibiotics and drainage of the shoulder joint. In our patient, joint drainage was performed through an anterior approach and abscess on the posterior aspect of scapula was drained through posterior scapular incision.

Although the early antibiotic treatment is related to a good prognosis, serious complications such as septic shock, osteomyelitis or relapse of the arthritis can occur. To prevent these complications, prompt diagnosis with identification of the causative organism and institution of the appropriate medical and surgical intervention is imperative to prevent joint destruction and possible permanent disability^4^. It is important for paediatricians to be aware of such unusual presentations of osteomyelitis and septic arthritis and maintain a high index of suspicion especially in children admitted to intensive care units.
